# The ENERTALK dataset, 15 Hz electricity consumption data from 22 houses in Korea

**DOI:** 10.1038/s41597-019-0212-5

**Published:** 2019-10-08

**Authors:** Changho Shin, Eunjung Lee, Jeongyun Han, Jaeryun Yim, Wonjong Rhee, Hyoseop Lee

**Affiliations:** 1 Encored Technologies, Seoul, Korea; 2 Department of Transdisciplinary Studies, Seoul National University, Seoul, Korea

**Keywords:** Energy supply and demand, Energy efficiency, Energy and behaviour

## Abstract

AMI has been gradually replacing conventional meters because newer models can acquire more informative energy consumption data. The additional information has enabled significant advances in many fields, including energy disaggregation, energy consumption pattern analysis and prediction, demand response, and user segmentation. However, the quality of AMI data varies significantly across publicly available datasets, and low sampling rates and numbers of houses monitored seriously limit practical analyses. To address these challenges, we herein present the ENERTALK dataset, which contains both aggregate and per-appliance measurements sampled at 15 Hz from 22 houses. Among the publicly available datasets with both aggregate and per-appliance measurements, 15 Hz was the highest sampling rate. The number of houses (22) was the second-largest where the largest one had a sampling rate of 1 Hz. The ENERTALK dataset is also the first Korean open dataset on residential electricity consumption.

## Abstract (summary)

**Table:** 

Measurement(s)	whole house energy consumption • appliance-by-appliance energy consumption • electrical energy
Technology Type(s)	mains electricity meter • plug-in individual appliance monitors • Gauge or Meter Device
Sample Characteristic - Environment	building
Sample Characteristic - Location	South Korea

Machine-accessible metadata file describing the reported data: 10.6084/m9.figshare.9874028

## Background & Summary

Sustainable energy has emerged as a global issue in the last twenty years, as exemplified by the Sustainable Development Goals adopted by the United Nations General Assembly in 2015, which include “affordable and clean energy” as one of 17 agenda items (https://sustainabledevelopment.un.org/). Kolter *et al*. made the REDD dataset publicly available to promote studies on energy disaggregation^1^, which is closely relevant to the sustainability issues. Many datasets on residential electricity consumption data have since been released^2–27^. However, although many datasets are now publicly available, the speed of innovation in the associated research fields, such as energy disaggregation, has been limited because of two main problems in the available datasets: low sampling rates and the small numbers of houses. As can be seen in Table 1, most of the datasets include only a few houses monitored at low sampling rates, especially at the appliance level, where the maximal sampling rate is 1 Hz. Furthermore, all the datasets except for Dataport and REFIT, were acquired from 10 or fewer houses. To address these two limitations, we provide a new dataset that contains data from 22 Korean houses, with both aggregate and appliance-level data acquired at a sampling rate of 15 Hz.

**Table 1:** Comparison of ENERTALK with other public datasets.

Dataset	Location	Duration	No. of houses (buildings)	No. of appliance instances	Sampling rate
REDD^1^	USA	3~19 days	6 houses	10~24 per house	15 kHz (aggregate only), 1 Hz (aggregate), 1/3 Hz (appliance)
BLUED^2^	USA	8 days	1 house	43 (on-off tag)	12 kHz (aggregate only)
Smart^3^	USA	3 months	3 houses	21~26 per house	1 Hz (aggregate and appliance)
Tracebase^4^	Germany	1 day	N/A	122	1 Hz (appliance only)
BERDS^5^	USA	1 year	1 house	4	20 sec. (aggregate and appliance)
AMPds^6^	Canada	1 year	1 house	19	1 min. (aggregate and appliance)
iAWE^7^	India	73 days	1 house	33	1 Hz (aggregate), 1 Hz or 6 sec. (appliance)
GREEND^8^	Austria/Italy	1 year	9 houses	9 per house	1 Hz (aggregate and appliance)
ECO^9,10^	Switzerland	8 months	6 houses	7~10 per house	1 Hz (aggregate and appliance)
PLAID^11^	USA	5 seconds	N/A	1074	30 kHz (appliance only)
COMBED^12^	India	1 month	6 buildings	200	30 sec. (aggregate and appliance)
DRED^13^	Holand	6 months	1 house	12	1 Hz (aggregate and appliance)
Dataport^14^	USA	4 + years	1200 + houses	~70 per house	1 Hz to 1 min. (aggregated and appliance)
UK-DALE^15–17^	UK	2.5 years	5 houses	5~54 per house	16 kHz (aggregate), 6 sec. (appliance)
AMPds2^18–21^	Canada	2 years	1 house	21	1 min. (aggregate and appliance)
REFIT^22–24^	UK	2 years	20 houses	9 per house	8 sec. (aggregate and appliance)
RAE^25^	Canada	72 days	1 house	24	1 Hz (aggregate and appliance)
I-BLEND^26,27^	India	52 months	7 buildings	N/A	1 min. (aggregate only)
ENERTALK^51^	Korea	29~122 days	22	1~7	15 Hz (aggregate and appliance)

Table 2 summarizes the ENERTALK dataset. For each of the 22 houses, we recorded the active and reactive power drawn by the entire house and individual appliances at every 1/15 of a second. We focused on the appliances that most Korean houses have: refrigerator, kimchi refrigerator, rice cooker, washing machine, and TV. The measurement periods differed for each house, from 29 days to 122 days. Our dataset is also, to the best of our knowledge, the first Korean electricity consumption dataset publicly available, thereby contributing to the regional diversification of globally available energy datasets. As one of the regional characteristics in the ENERTALK dataset, our dataset has measurements for kimchi refrigerators, which is a special type of refrigerator for the storage and fermentation of kimchi, a staple of Korean cuisine. This type of regional characteristic is important to understand regional differences in electricity consumption patterns, which impacts global energy consumption and sustainability targets^28^.

**Table 2:** Summary of the 22 houses.

House code	Start date	End date	Duration (days)	Refrigerator	Kimchi refrigerator	Rice cooker	Washing machine	TV	Microwave	Water-purifier
00	2016-11-01	2017-01-31	91	O	O	O	O	O	O	O
01	2016-10-01	2017-01-31	122	X	O	O	O	O	X	X
02	2016-10-01	2016-10-31	30	O	X	O	O	O	X	X
03	2016-10-01	2017-01-31	122	X	O	X	O	X	X	X
04	2016-09-01	2016-11-30	90	O	X	O	O	O	X	X
05	2016-09-03	2016-10-31	58	O	O	O	O	O	X	X
06	2016-09-01	2016-10-15	44	O	O	X	O	O	X	O
07	2016-12-01	2017-01-31	61	X	O	X	X	O	X	X
08	2016-12-01	2017-01-31	61	X	O	O	O	O	X	X
09	2016-10-01	2017-01-31	122	O	X	O	X	O	O	X
10	2016-10-01	2017-01-31	122	O	O	X	X	X	X	X
11	2017-04-01	2017-04-30	29	X	X	O	X	O	X	X
12	2016-10-01	2017-01-31	122	O	O	O	O	O	X	X
13	2016-11-02	2017-01-31	90	X	O	O	X	O	X	X
14	2016-10-01	2017-01-20	111	O	X	X	X	X	X	X
15	2017-03-15	2017-04-30	46	O	X	X	X	O	X	X
16	2016-09-01	2016-11-15	75	O	X	X	X	X	X	X
17	2016-11-03	2017-01-31	89	O	O	O	O	O	X	X
18	2016-09-01	2016-10-19	48	O	O	X	O	O	X	X
19	2016-09-01	2016-10-31	60	O	X	X	X	O	X	X
20	2017-03-01	2017-04-30	60	O	O	O	X	X	X	X
21	2016-12-01	2017-01-31	61	X	O	O	O	O	X	X

Our dataset was originally designed for energy disaggregation research. However, ENERTALK can be used for a variety of research fields, as shown in Table 3. Energy disaggregation involves estimating each individual appliance’s energy usage from the total aggregated power consumption measurements. Initially proposed by Hart (1992)^29^, energy disaggregation is still an active area of research^30–37^. Data on disaggregated energy usage enables more direct feedback on consumers’ energy consumption behaviors. Neenan and Robinson showed that energy breakdown information can lead consumers to energy-saving behaviors that improve energy consumption effciency by 15%^38^. Non-intrusive load monitoring (NILM) can also be used to detect malfunctioning appliances, design energy incentives, manage demand-response, etc.^39,40^.

**Table 3:** Main research fields that are based on the energy consumption datasets.

Research field	Description
Energy disaggregation (Non-Intrusive Load Monitoring; NILM)	Estimation of the power consumption of an individual appliance from the aggregated power consumption^29–37^
User segmentation	Categorization of users based on energy consumption behavior^41–44^
Electricity consumption pattern analysis	Exploratory data analysis on residential electricity consumption patterns^42,45,46^
Power consumption forecasting	Prediction of power consumption based on power consumption history^47–49^

In another line of research, user segmentation is the problem of categorizing households based on their energy consumption patterns^41–44^. For example, Kwac *et al*. clustered households based on hourly energy consumption data that showed typical energy usage patterns depending on the hour of the day^41^. Such clustering revealed certain lifestyle features of households, and the segmentation could be used for targeted demand-response programs. User segmentation research can also be used for services such as targeted marketing and promotions based on household types.

Electricity consumption pattern analysis is another research field that relies heavily on residential electricity consumption data^42,45,46^. For instance, Kavousian *et al*. analyzed electricity consumption data in relation to climate, building characteristics, appliance stock, and occupant behaviors^45^. This type of analysis is important for policy-making, and energy-efficiency programs have been adapted using consumption patterns thus identified.

Another important research area is electricity consumption prediction, in which future electricity consumption is predicted based on individual electricity consumption histories^47–49^. For example, Marvuglia and Messineo studied short-term forecasting (1 hour in advance) of residential electricity consumption using recurrent neural networks^49^. Such research can be especially helpful for demand response programs because electricity consumption predictions can guide the timing of demand-response programs^50^.

## Methods

**Fig. 1:**
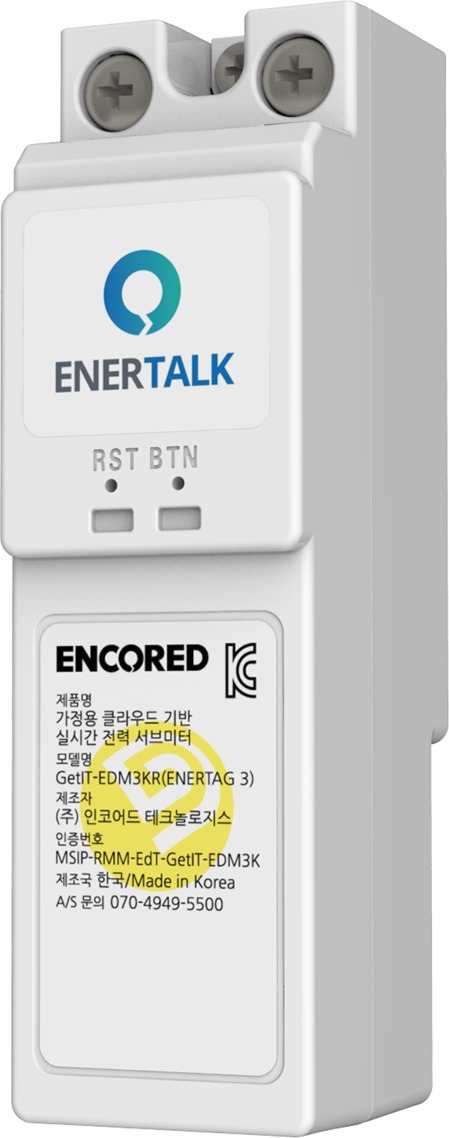
ENERTALK.

**Fig. 2:**
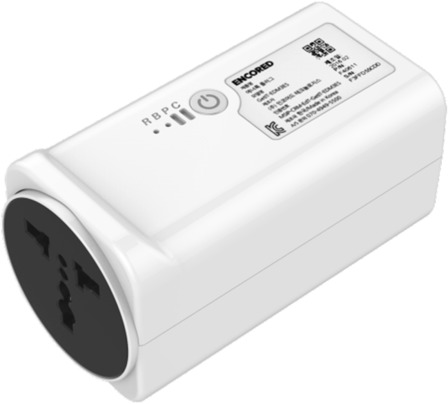
ENERTALK PLUG.

The electricity consumption in each house was measured with off-the-shelf smart meters: ENERTALK and ENERTALK PLUG. We used ENERTALK to measure the aggregate power consumption of the whole house, and we used ENERTALK PLUG to measure the power consumption of individual appliances. Within each house, one ENERTALK and one or more ENERTALK PLUGs (for one or more appliances) were installed. Figures 1 and 2 show the devices, and Table 4 provides the specifications for the device hardware. In addition to the information provided in the specification, ENERTALK and ENERTALK PLUG were calibrated to guarantee the error rate of one percent or below. This is in accordance with IEC 62053-21 standard. The ENERTALK devices were installed in each house’s fuse box to measure aggregate power consumption. In order to measure the electricity consumption of appliances, ENERTALK PLUG devices were plugged into the AC outlets, and the appliances were plugged into the ENERTALK PLUG devices.

**Table 4:** Measurement device specifications.

	Enertalk	Enertalk Plug
MCU CPU	STM (ARM Cortex-M0)	STM (ARM Cortex-M0)
MCU Frequency	48 MHz	48 MHz
Flash (KB)	128	256
SRAM (KB)	16	32
Wi-fi connectivity	802.11 b/g/n, single band (2.4 G)	802.11 b/g/n, single band (2.4 G)
Voltage range	100–240 V AC	100–240 V AC
Frequency range	50/60 Hz	50/60 Hz
Operating Temperature	−20 °C~50 °C	−20 °C~50 °C

**Fig. 3:**
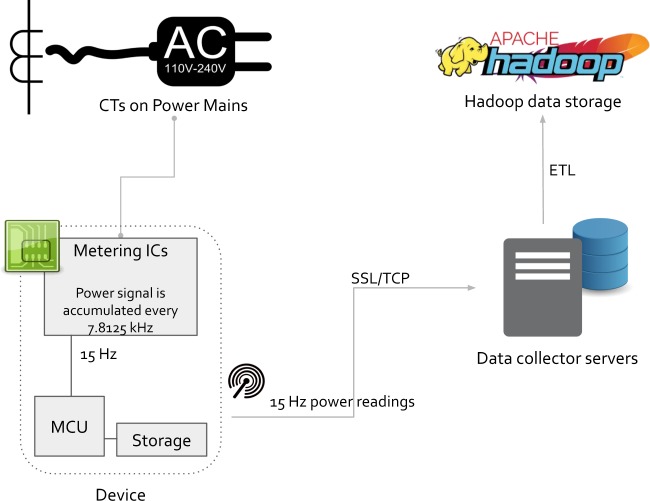
Data collection schematic.

A schematic of the data collection system using ENERTALK is depicted in Fig. 3. Active power and reactive power records were generated by currents measured by the current transformer clamp. The ENERTALK and ENERTALK PLUG devices have voltage ranges of 100–240 V. Power signals accumulated every 7.8125 kHz at the metering integrated circuits, and these signals were down-sampled to 15 Hz, processed, and saved in the device storage by the microcontroller unit. The 15 Hz power readings collected by the smart meters were sent to our cloud data collection servers via SSL/TCP, and the data collection servers converted the received data into a structured form. After the data were successfully transformed, the data collection servers saved the data in a Hadoop database. The final dataset was saved as Parquet files after pre-processing to remove unnecessary or private information. The monitored houses were mainly occupied by employees of Encored, Inc., and/or acquaintances of the employees. To assist in the collection and sharing of the ENERTALK dataset, these people kindly agreed to install metering devices in their houses and to allow public access of the measured data. In each house, the occupants selected the appliances to be recorded. When choosing appliances to record, occupants were asked by the researchers to prioritize those household appliances that are widely used in Korea. The resulting selections are shown in Table 2. In the table, it can be noticed that heating, cooling, and lighting are missing. The electricity loads of the three are significant in many countries, but they were not included for the following reasons. As for the heating, typically it is done with gas in Korea. As for the cooling, the measurement campaign happened to occur excluding the hot summer season, and therefore cooling devices were not in use. As for the lighting, each household had many lighting devices that it was impossible to install ENERTALK PLUGs for all and measuring one or few lighting devices did not seem helpful, either.

## Data Records

The ENERTALK dataset is publicly available for download from figshare^51^. It uses a data format similar to that used by the well-known NILM datasets REDD^1^ and UK-DALE^15^. The ENERTALK dataset includes 22 directories, one per house. The directories are named using a two-digit integer number, such as “00” or “01”, such that each two-digit number represents a specific house. Each directory holds a set of subdirectories that contain Parquet files for the daily aggregate and appliance-level data. The naming convention for these subdirectories is “<yyyy> <mm> <dd>” (e.g. “20161124” for November 24, 2016). The Parquet files are named “<two digit integer>_<appliance name>.parquet.gzip” (e.g. “01_fridge.parquet.gzip”). In these names, the two-digit integer is uniquely associated with a distinct measuring device in a house. Each Parquet file consists of three columns: “timestamp”, “active_power”, and “reactive_power”. The “timestamp” column contains Unix timestamps in milliseconds, such that 1000 corresponds to one second. The “active_power” column represents active power in watts, and the “reactive_power” column represents reactive power in VAR (volt-ampere reactive) units.

## Technical Validation

Each of the 22 Korean houses provided 29~122 days of aggregate and appliance-level power consumption data, for the appliance categories summarized in Table 2.

**Fig. 4:**
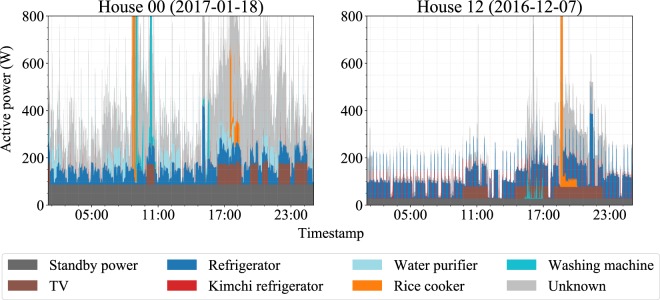
Data snippets from two houses: color-coded according to the appliance type.

Figure 4 presents two data snippets, for house 00 and house 12, respectively, that show the itemized power consumption patterns for one day in the two houses. At the appliance level, the refrigerator, kimchi refrigerator, and water purifier generally operate all day. In contrast, other typical appliances, such as the TV and washing machine, are mainly turned on only when the occupants used these appliances, and energy consumption by these appliances is therefore closely related to the lifestyles of the occupants. For example, in some houses, washing machines tend to be used on weekends because the occupants do laundry on when they are not at work. As another consideration, although rice cookers are generally used at mealtime, they are also maintained in the “on” state when used in “keep warm” mode. The data also show “unknown” power consumption-the difference between the aggregate power consumption and the sum of the appliance power consumptions-even though we tried to measure as many appliances as possible.

**Fig. 5:**
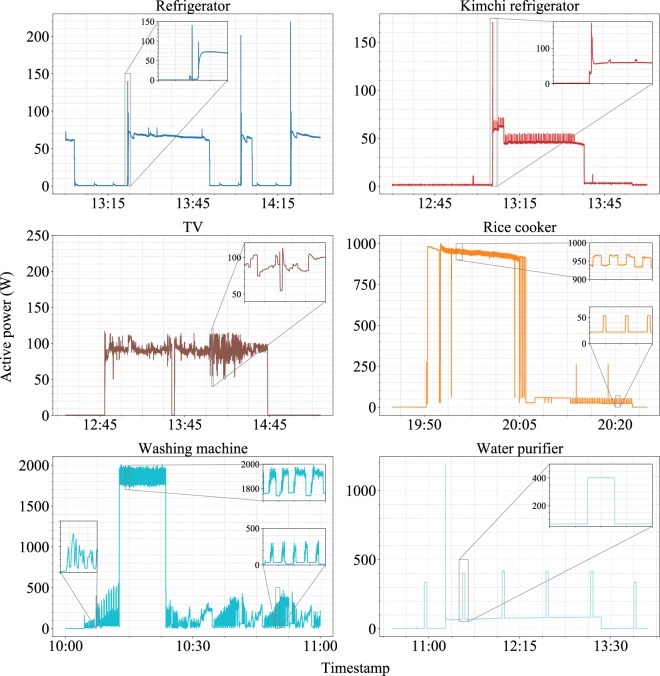
Data snippets from six different appliances.

Figure 5 shows the typical electricity consumption patterns for each appliance, which are clearly distinguishable from one another. The refrigerator and kimchi refrigerator patterns show the periodicity of power consumption for cooling. When their compressors start to operate, consumption tends to overshoot initially, followed by box-shaped power consumption. Although the refrigerator and kimchi refrigerator exhibit similar power consumption patterns in this data, kimchi refrigerators are known to consume less electricity than refrigerators. The TV consumption patterns show fluctuations in the “on” state. These fluctuations originate from changes in the activation of screen pixels. TV consumption patterns are known to be almost the same if the same TV model plays identical TV programs^36^. The washing machine and rice cooker have multiple operation states, resulting in multiple power consumption modes. The rice cooker in the figure shows two distinct power consumption patterns that correspond to “cooking” mode and “keep warm” mode. The rice cooker in the “cook” mode consumes more power (approximately 1000 W) in a rectangular sawtooth pattern, whereas in the “keep warm” mode, the rice cooker consumes less power (20–50 W), and the consumption pattern shows a stepped shape. The washing machine pattern shows three laundry stages: pre-wash, wash, and rinse. In the pre-wash stage, the power consumption oscillates with increasing amplitude, followed by a wavering sawtooth above approximately 1800 W for the wash stage. In the rinse stage, oscillations with periodic peaks are repeated in a very fast cycle. The water purifier consumption pattern shows two types of box shapes that appear periodically: one related to water heating, which shows power consumption of 400 W, and the other related to water cooling, which shows power consumption of 80 W; both signals show overshooting when the water purifier begins to operate.

Considering Figs 4 and 5 together, we can trace the power consumption patterns of each appliance after aggregation. For example, we can identify the power consumption of the kimchi refrigerator in the periodic spikes in the aggregated power consumption. As another example, we can estimate rice cooker use from the large amount of aggregated power consumption shown in the morning and evening.

**Fig. 6:**
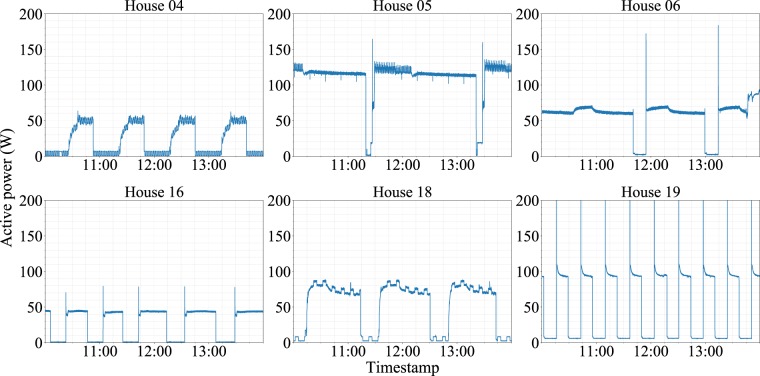
Data snippets from six refrigerators from six different houses. All of the six refrigerators show distinct patterns that are visually distinguishable from one another.

However, the electricity consumption patterns of these appliances are not always the same. Depending on the appliances’ inner components, their electricity consumption patterns can be very different. For instance, Fig. 6 shows the power consumption patterns of six different refrigerators, and each pattern shows a distinctive shape in terms of overshooting, “on” state consumption, and duration, implying that measurements from a large number of devices in the same type of appliance category may be necessary to build generalizable NILM algorithms. In widely used datasets such as REDD and UK-DALE, the number of measuring devices in the same appliance category is quite limited. In contrast, the dataset presented here contains more than ten devices for each appliance, except for the microwave and water purifier.

**Fig. 7:**
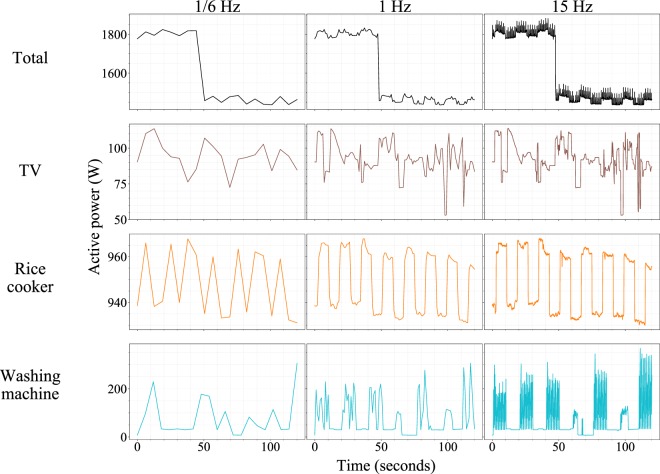
Power consumption measurements at sampling rates of 1/6, 1, and 15 Hz. The TV, rice cooker, and washing machine show distinct and visually distinguishable patterns at 15 Hz, but the patterns become less distinguishable at 1 Hz and become visually comparable at 1/6 Hz. Each plot shows 120 seconds of duration; 1/6 and 1 Hz data were generated by down-sampling (taking the first measurements of every 6 seconds and 1 second, respectively).

A high sampling rate can help to maximize the utility of the appliance-specific consumption patterns described above. Armel *et al*. have shown that the higher the sampling rate, the more appliances can be distinguished in the power consumption patterns^52^. Shin *et al*. also reported that higher sampling rates can be beneficial for empirical energy disaggregation because the appliance signatures become more visible as the sampling rate increases^53^. In line with these studies, Fig. 7 shows how the power consumption patterns change when the sampling rate increases from 1/6 Hz (one sample each 6 seconds), to 1 Hz, and then to 15 Hz. The two lower rates correspond to the sampling rates of UK-DALE and REDD, respectively. Although the overall shapes of the patterns are similar, the rice cooker and washing machine data are conspicuous at the 15 Hz sampling rate, whereas the shapes start to become ambiguous as the sampling rate is reduced to 1 Hz and 1/6 Hz. At 1/6 Hz, the power consumption patterns of most appliances are elusive, limiting the ability to disaggregate the appliances. Although appliances sampled at the 1 Hz rate are more distinguishable than those sampled with the 1/6 Hz rate, the micro-patterns are much clearer in the 15 Hz data than the 1 Hz data. For example, the oscillation frequency and amplitude are clearly visible in the washing machine consumption pattern acquired at the 15 Hz rate; however, the 1 Hz and 1/6 Hz data do not show these frequency and amplitude characteristics in such detail.

**Fig. 8:**
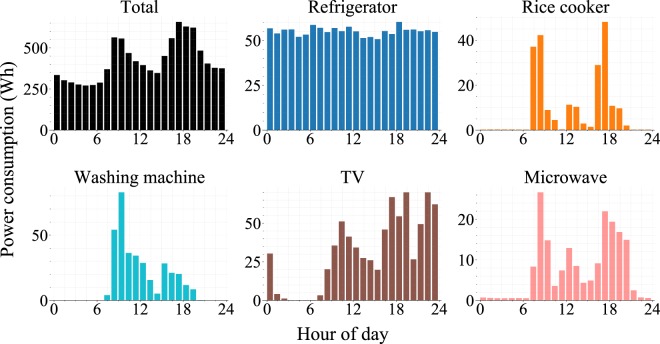
Hourly distribution of average power consumption (house 00).

The ENERTALK dataset is not only useful for energy disaggregation research, but also for lifestyle analyses. Figure 8 shows house 00’s hourly power usage distribution over 24 hours. Total power consumption is concentrated in the morning and evening, which would be a typical pattern for a house occupied by someone who is a daytime worker. As expected, the refrigerator’s power consumption is evenly distributed over the 24 hours because the cooling system in the refrigerator operates periodically. Consumption associated with the cooking appliances (rice cooker, microwave) is concentrated in the morning and evening, indicating that the occupants took meals at home at these times. The patterns also show that the washing machine was mainly used in the morning, and the TV was mainly used late at night. These types of lifestyle analyses based on electricity consumption patterns can be used for user segmentation^41,42^.

**Fig. 9:**
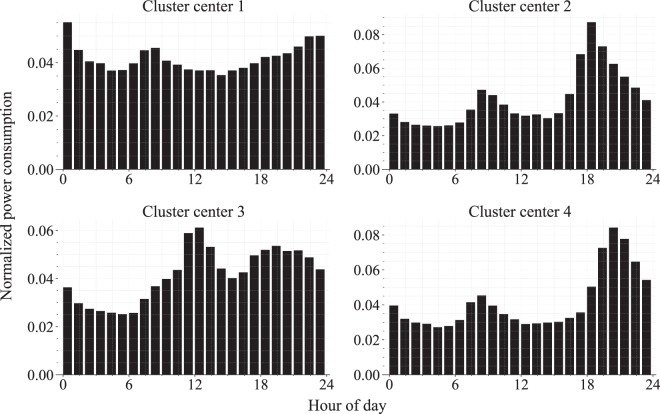
Top four cluster centers found with adaptive K-means clustering on the normalized data. Each cluster center shows one of the most typical patterns of the daily total power consumption patterns.

As an example of total usage analysis, adaptive K-means clustering was conducted on normalized daily total usage based on the method presented by Kwac *et al*.^41^. In their study, they convert hourly measurements into a 24-hour daily consumption profile, in which a house’s power consumption in one day is represented as a 24-dimension vector; they then normalized each daily profile with the house’s total consumption of 24-hour and applied clustering methods to the normalized daily profiles to find typical daily consumption patterns. Following this method, we down-sampled the aggregate power consumption data in our dataset into hourly measurements and applied Kwac *et al*.’s data processing method and clustering. Figure 9 shows the top four cluster centers from the results of the adaptive K- means clustering, with cluster numbers assigned in the order of the number of data points belonging to each cluster. Because the data collection targets were mainly the houses where office employees lived, the electricity consumption patterns in cluster centers 1, 2, and 4 were closely related to typical office hours (09:00–18:00 or 10:00–19:00, depending on the type of working hours). The electricity consumption patterns in clusters 1, 2, and 4 show increases in the rush hour and in the hours after work. Thus, clusters 1, 2, and 4 appear to represent different typical working days that depend on the commute time, the ratio of standby power consumption, and other lifestyle characteristics. Cluster 3 appears to represent power consumption patterns on the weekend, given that power consumption is concentrated at lunchtime or in the evening rather than at the commute time.

**Fig. 10:**
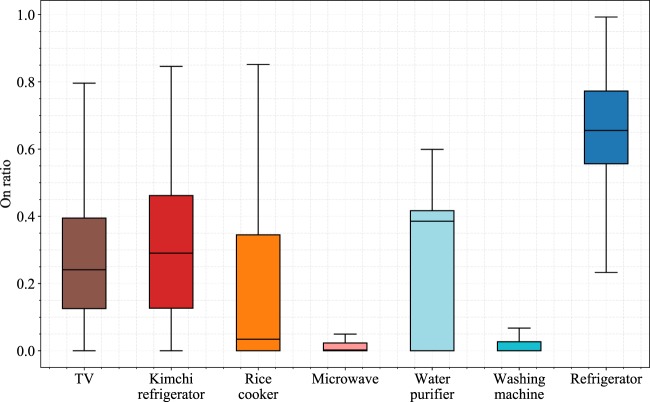
Box plot of daily on-ratio for all 22 houses. For each sample, an appliance was considered to be on if the appliance’s active power was above 15 W.

The extent to which people use each appliance can help to determine the importance of each appliance in that person’s lifestyle. Figure 10 shows how long each appliance is used on average during the day. The TV shows an average on ratio of slightly over 20%, which represents an average of approximately 5 hours per day of TV usage. The refrigerator and kimchi refrigerator show high on ratios because their periodic cooling systems work all day long. The rice cooker and water purifier show much higher on ratios than people generally expect because the rice cooker’s “keep warm” mode and the water purifier’s heating and cooling processes operate periodically. The washing machine and microwave show relatively low on ratios because these appliances are not frequently used, and they are used for only a fixed amount of time.

## Usage Notes

**Fig. 11:**
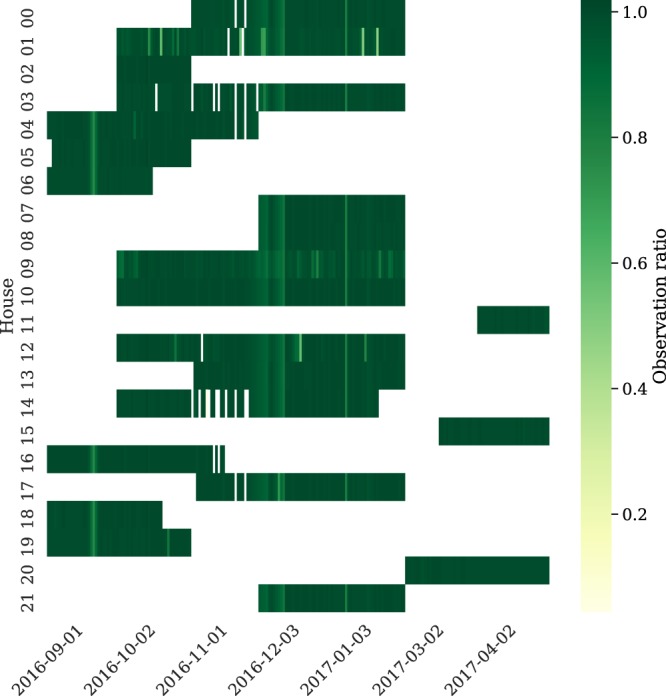
Heatmap of daily observation ratio for all 22 houses. White spaces represent the days with no observation (no data collection). The observation ratio was calculated by dividing the number of collected data samples by the maximum number of samples collectable in a day (1,296,000 samples).

When using time series data such as the ENERTALK dataset, the approach to handling missing values is the most difficult and practical problem. In our data collection process, missing values occurred owing to diverse causes, including network problems and measurement device errors. As shown in Fig. 11, the ENERTALK dataset was acquired with nearly perfect observation ratios for most days. However, imputation methods were still required to handle the missing values properly^54^.

In the NILM literature, forward filling methods are generally used for this purpose^34,37^, whereby missing values are filled with the closest of the previously observed values. Other methods such as linear interpolation and EM algorithms can be used to address missing values, as appropriate for the researcher’s intention and task.

**Fig. 12:**
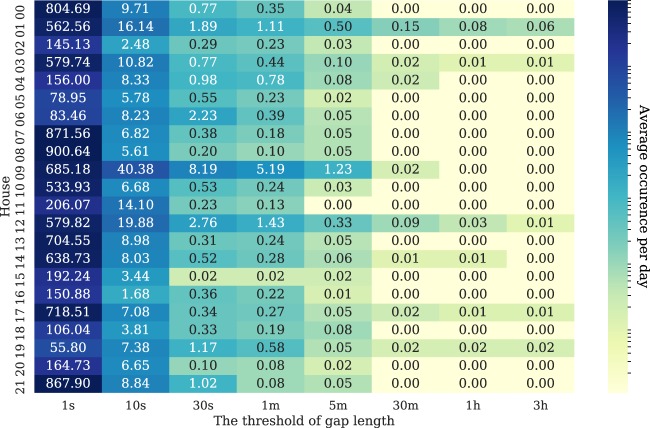
Average daily occurrence of data gaps for all 22 houses. Gap thresholds of 1 s, 10 s, 30 s, 1 min, 5 min, 30 min, 60 min, and 180 min were considered.

One of the considerations when we pre-process data is the length of each sequence of missing values because this length can affect the suitability of the imputation method. For example, although short sequences of missing values can be effectively filled using forward filling or linear interpolation, long sequences should perhaps be discarded for that interval. In^37^, NILM researchers defined a “gap” as an interval between any pair of consecutive samples where the time elapsed between them is larger than a predefined threshold. The average daily occurrence of gaps in the ENERTALK dataset is shown in Fig. 12, with different predefined thresholds.

The number of gaps decreased exponentially as the threshold increased from one second to one minute. These results could be used to determine the imputation method. We thus used linear interpolation for values missing for less than thirty seconds and discarded values missing for over thirty seconds. The number of gaps lasting over thirty seconds decreased exponentially, and interpolation for such long sequences is meaningless.

**Fig. 13:**
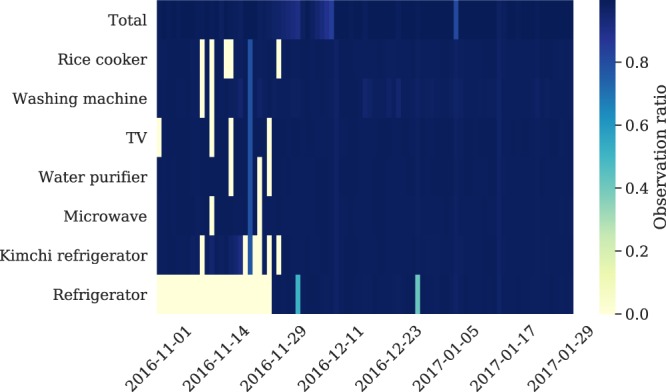
Observation ratio of House 00. Apparently, where data collection occasionally failed. In particular, the refrigerator data collection for the first one month completely failed.

Data timestamp alignment is another challenge when using aggregate and appliance-level data. First, milliseconds are not easy to align because the timestamp in each appliance is recorded based on measurements in each individual ENERTALK device without considering other appliances connected to such devices. A solution for this problem is to discretize milliseconds into 15 bins and use the discretized values to align the aggregate and appliance-level data, and Fig. 4 was plotted using the solution. The second problem is that the missing values from each dataset require a choice as to whether to discard only the affected appliance data for that day or to discard all data for that day, as exemplified in Fig. 13. These cases can be handled in different ways depending on the purpose of the research. For example, when using the ENERTALK dataset to disaggregate TV power consumption from aggregated consumption, researchers can select those days with high observation ratios of total and TV power consumption while ignoring other appliances.

Our github repository (https://github.com/ch-shin/ENERTALK-dataset) contains the basic tools for handling problems such as missing values and misalignments. In addition to the basic tools, we provide visualization notebook and an NILMTK converter^37^ specifically designed for NILM researchers. The codes are provided only as a default option, and the users should modify or rewrite the codes according to the purpose of using the dataset.

## Data Availability

The scripts used for pre-processing and visualization in Data Records and Usage Notes are available at our github (https://github.com/ch-shin/ENERTALK-dataset). Unfortunately, the codes for collecting and storing data are the private property of the enterprise (Encored Inc.) and cannot be opened.
